# Computational Analysis of Missense Variants in the Human Transmembrane Protease Serine 2 (*TMPRSS2*) and SARS-CoV-2

**DOI:** 10.1155/2021/9982729

**Published:** 2021-10-19

**Authors:** Asmae Saih, Meryem Bouqdayr, Hanâ Baba, Salsabil Hamdi, Samya Moussamih, Houda Bennani, Rachid Saile, Lahcen Wakrim, Anass Kettani

**Affiliations:** ^1^Virology Unit, Immunovirology Laboratory, Institut Pasteur du Maroc, 20360 Casablanca, Morocco; ^2^Laboratory of Biology and Health, URAC 34, Faculty of Sciences Ben M'Sik Hassan II University of Casablanca, Morocco; ^3^Environmental Health Laboratory, Institut Pasteur du Maroc, 20360 Casablanca, Morocco; ^4^Immunology and Biodiversity Laboratory, Faculty of Sciences Ain Chock, Hassan II University of Casablanca, Morocco

## Abstract

The human transmembrane protease serine 2 (*TMPRSS2*) protein plays an important role in prostate cancer progression. It also facilitates viral entry into target cells by proteolytically cleaving and activating the S protein of the severe acute respiratory syndrome coronavirus 2 (SARS-CoV-2). In the current study, we used different available tools like SIFT, PolyPhen2.0, PROVEAN, SNAP2, PMut, MutPred2, I-Mutant Suite, MUpro, iStable, ConSurf, ModPred, SwissModel, PROCHECK, Verify3D, and TM-align to identify the most deleterious variants and to explore possible effects on the *TMPRSS2* stability, structure, and function. The six missense variants tested were evaluated to have deleterious effects on the protein by SIFT, PolyPhen2.0, PROVEAN, SNAP2, and PMut. Additionally, V160M, G181R, R240C, P335L, G432A, and D435Y variants showed a decrease in stability by at least 2 servers; G181R, G432A, and D435Y are highly conserved and identified posttranslational modifications sites (PTMs) for proteolytic cleavage and ADP-ribosylation using ConSurf and ModPred servers. The 3D structure of *TMPRSS2* native and mutants was generated using 7 meq as a template from the SwissModeller group, refined by ModRefiner, and validated using the Ramachandran plot. Hence, this paper can be advantageous to understand the association between these missense variants rs12329760, rs781089181, rs762108701, rs1185182900, rs570454392, and rs867186402 and susceptibility to SARS-CoV-2.

## 1. Introduction

Transmembrane protease serine 2, also called *TMPRSS2*, is an androgen-regulated gene that is located at human chromosome 21q 22.3, approximately extends 43.59 Kb in length, and contains 14 exons [1]. *TMPRSS2* is locally expressed in many tissues, comprising the prostate, bile duct, breast, kidney, colon, pancreas, ovary, stomach, salivary gland, and lung [1]. The full-length *TMPRSS2* cDNA encodes a protein of 492 amino acids, with a type II transmembrane domain, a receptor class A domain (LDLRA, aa 113-148), a scavenger receptor cysteine-rich domain (SRCR, aa 149-242), and a serine protease domain (aa, 255-492) [2].

To date, physiological roles of the transmembrane protease serine 2 are unknown, but it participates in many biological processes such as digestion, fertility, blood coagulation, tissue remodeling, inflammatory responses, tumor cell invasion, and apoptosis [2]. *TMPRSS2* in turn plays an essential role in prostate tumorigenesis via the proteolytic activation of the protease-activated receptor 2 (PAR-2) [3, 4]. A study by Magi-Galluzzi et al. about prostate cancer (Pca) revealed that TMPRSS2-ERG fusion was significantly correlated with ethnicity and geography (50% of Caucasians, 31.3% African-Americans, and 15.9% of Japanese patients) [5]. Another study by Kong et al. explored the association between the TMPRSS2-ERG gene fusion and clinicopathological characteristics and reported that no significant correlation was observed between the TMPRSS2-ERG gene fusion and clinical parameters [6].

Recently, it has been shown that SARS-CoV-2 engages angiotensin-converting enzyme 2 (ACE2) as the entry receptor and uses *TMPRSS2* for S protein priming [7]. Overall, SARS-CoV-2 has been determined by four types of structural, i.e., spike (S), envelope (E), membrane (M), and nucleocapsid (N) proteins, and accessory proteins like ORF3a, ORF7a, ORF8, ORF9, and ORF10 [8, 9].

The S protein is composed of an extracellular N-terminal associated with S1 essential for binding the receptor and a C-terminal labelled S2 that is used for membrane fusion. The envelope E protein is composed of a hydrophilic amino acid terminus (7-12 AA), the transmembrane hydrophobic domain, and a long C-terminal domain that are essential for viral assembly and maturation. The M protein is composed of a hydrophilic C-terminal and amphipathic N-terminal which are needed for viral assembly. The N protein consists of an N-terminal RNA domain (NTD) and a C-terminal dimerization (CTD) domain separated by a serine-rich linker region that are essential for viral entry and assembly [8, 9]. As *TMPRSS2* is expressed in bronchial and lung cells, it can therefore facilitate entry of SARS-CoV-2 into host cells by cleaving the ACE2 receptor at arginine 697-716 positions [2]. The *TMPRSS2* protein is responsible for the proteolytic cleavage of the viral spike protein (S) [10]. Several studies have demonstrated the existence of three residues of catalytic triad of *TMPRSS2*, namely, His 296, Asp 345, and Ser 441, that play a crucial role in the involvement of molecular complex between *TMPRSS2* and viral spike protein S and consequently SARS-CoV-2 [10].

Recent studies show the existence of unique variants in *TMPRSS2* (p. Val160Met, p. Gly181Arg, p. Arg240Cys, p. Pro335Leu, p. Gly432Ala, and p. Arg435Tyr) that can alter the efficiency of *TMPRSS2* and might influence susceptibility to SARS-CoV-2 [11].

Taking into account all these considerations, this article is aimed at elucidating the plausible effect of *TMPRSS2* genetic missense variants in structure, stability, and functions of *TMPRSS2* using different publicly available bioinformatics algorithms. The use of a wide array of pathogenicity tools like SIFT, PolyPhen2.0, PROVEAN, SNAP2, and PMut provides consistent results. Also, stability, conservation, and flexibility approaches using bioinformatics tools, namely, I-Mutant Suite, MUpro, iStable, STRUM, CUPSAT, ConSurf, ModPred, and FlexPred, will aid comprehending the mutation effect on *TMPRSS2* protein [12–15].

## 2. Materials and Methods

### 2.1. Datasets

The amino acid sequence of the *TMPRSS2* gene was obtained in FASTA format from UniProt databases (UniProt ID: O15393) (https://www.uniprot.org). All the variants of the *TMPRSS2* gene were collected from *Ensembl Genome Browser* (https://www.ensembl.org/Homo_sapiens/Gene/Variation_Gene/Table?db=core;g=ENSG00000184012;r=21:41464305-41531116). A total of 392 missense variants were mapped in the human *TMPRSS2* gene, but we limited our study to those SNPs who provide explanations for genetic susceptibility to COVID-19; therefore, six variants remained.

### 2.2. Functional Analysis of Human *TMPRSS2* Missense Variants

SIFT (Sort Intolerant from Tolerant) is a sequence homology-based algorithm that predicts tolerable and intolerable change in protein function caused by the substitution in amino acid sequence, which is available at https://sift.bii.a-star.edu.sg/ [16]. A substitution is predicted to be “deleterious” if the prediction score ranges from 0 to 0.05 and “tolerable” if the prediction score is greater than or equal to 0.05 [17]. PolyPhen2.0 (Polymorphism Phenotyping v2) is a web server that uses physical and comparative considerations to estimate the effect of substitution of an amino acid on protein function and structure, which is available at https://genetics.bwh.harvard.edu/pph2/ [18]. PROVEAN (Protein Variation Effect analyzer) is an algorithm that predicts the possible impact of the substitution of amino acid, based on the alignment score approach, which is available at http://provean.jcvi.org/[50]. SNAP2 (Screening of nonacceptable Polymorphism 2) is a bioinformatics tool that uses the annotations from the protein mutant database (PMD) to predict the changes due to the nsSNPs on protein function, which is available at https://rostlab.org/services/snap/ [19]. PMut (http://mmb.irbbarcelona.org/PMut) is a tool, developed based on a neural network classification method, which uses both sequence conservation and physicochemical properties to predict disease-associated mutations [20]. MutPred2 (http://mutpred.mutdb.org/) is a machine learning approach that predicts the molecular cause of disease-related amino acid change. MutPred2 comprises functional, structural, and evolutionary properties including secondary structures, posttranslational modification (PTM), and metal binding [21].

### 2.3. Structural Analysis of Human *TMPRSS2* Missense Variants

#### 2.3.1. Protein Stability

I-Mutant Suite is a web server based on a support vector machine developed to predict the stability change of the mutated protein sequence or structure when available. I. Mutant predicts if a given mutation increases (∆∆*G* > 0) or decreases (∆∆*G* < 0) the protein stability and is available at https://gpcr2.biocomp.unibo.it/cgi/predictors/I-Mutant3.0/I-Mutant3.0.cgi [22]. MUpro (http://mupro.proteomics.ics.uci.edu/) is an online server used to predict the stability change for single-site mutations, depending on the structure or sequence information. MUpro adopts a support vector machine as an estimator to calculate the ∆∆*G* value and evaluate the direction of stability change of the protein [23]. iStable (Integrated Predictor for Protein Stability Change Upon Single Mutation) (http://predictor.nchu.edu.tw/istable/indexSeq.php) is a web server that uses a support vector machine as an integrator to predict the value of free energy stability (DDG). DDG < 0 for each variant was considered as decreasing the stability of the protein [24, 25]. STRUM (https://zhanglab.ccmb.med.umich.edu/STRUM/) is a tool based on a gradient boosting regression approach to predict the effect of a site mutation on stability. ∆∆*G*<0 means the variant decreases the protein stability and vice versa [26]. CUPSAT (Cologne University Protein Stability Analysis Tool) is a web server that combines both structural environment-specific atoms and torsion angles to predict protein stability changes upon point mutations, which is available at http://cupsat.tu-bs.de/ [27].

#### 2.3.2. Identification of Conserved Residues and Sequence Motifs

Clustal Omega, a bioinformatics program, was used to align multiple homologous proteins or DNA/RNA sequences. It uses both the older clustalX and clustalW for multiple sequence alignment. Clustal Omega is available at https://www.ebi.ac.uk/Tools/msa/clustalo/ or can be used from the command line [28]. Jalview is a freely available system (https://www.jalview.org), which was used for visualization, editing, figure generation, and analysis of molecular sequences, alignment, and structures, provided by the European Bioinformatics Institute (EBI) and the University of Dundee [29].

#### 2.3.3. Evolutionary Phylogenetic Analysis of *TMPRSS2*

ConSurf (https://consurf.tau.ac.il/) is an in silico tool that uses an empirical Bayesian method for estimating the degree of evolutionary conservation of an amino acid in macromolecules (protein or nucleic acid). The conservation grades are ranged from 0 to 9, where 1–4 score is variable, 5–6 score is intermediate, and 7–9 score is conserved [30].

#### 2.3.4. Prediction of Posttranslational Modification

ModPred (http://www.modpred.org/), a web server, was developed to predict posttranslational modification sites (PTMs) such as acetylation, methylation, N-linked glycosylation, N-terminal acetylation, phosphorylation, SUMOylation, and ubiquitination. As a PTM predictor, ModPred estimates the overall propensity of a particular amino acid to be changed [31].

#### 2.3.5. Protein Flexibility

FlexPred (http://flexpred.rit.albany.edu/) a bioinformatic program uses two sequence-derived information and solvent accessibility to evaluate residue positions involved in conformational switches. FlexPred classifies amino acid residues into rigid or flexible [32]. PredyFlexy (https://www.dsimb.inserm.fr/dsimb_tools/predyflexy/) is an online tool, which was used to predict protein flexibility. PredyFlexy adopts the X-ray *B*-factors and the root mean square fluctuations (RMSF) for predicting the flexibility of local protein structures [33]. RaptorX Property (http://raptorx.uchicago.edu/StructurePropertyPred/predict/) is a web-based server implementing a powerful machine learning method named deepCNF (deep convolutional neural fields) to evaluate and calculate protein secondary structure, disorder regions, and solvent accessibility [34].

#### 2.3.6. Secondary Structure

*PredictProtein* (https://predictprotein.org/) is an automatic server that uses FASTA amino acid sequence as input and predicts protein structure such as secondary structure, solvent accessibility, disulfide bonds, transmembrane helices, strands, coiled-coil regions, and disordered regions, and function [35].

### 2.4. Modeling

*Swiss-Model*(https://swissmodel.expasy.org/), an automated server, was used for predicting the three-dimensional structure of proteins. Using FASTA amino acid sequence as input, the Swiss-Model server searches for templates and/or for model building. It gives the best models with sequence identity higher than 30% [36]. *ModRefiner* (https://zhanglab.ccmb.med.umich.edu/ModRefiner/), an online server, was used for high-resolution protein structure refinement. ModRefiner adopts two separate phases: firstly, it starts from C-alpha trace and main chain hydrogen-bonding networks. Secondly, the side chain is added onto the backbone conformation with the guide of a composite of physics and knowledge-based force fields [37]. *PROCHECK* (https://servicesn.mbi.ucla.edu/PROCHECK/) is a web-based tool for assessing the quality of protein structure. Its outputs contain a large number of plots including the Ramachandran plot [38]. *Verify3D*, a freely available online server (https://servicesn.mbi.ucla.edu/Verify3D/), was used to verify the quality assessment of protein models with three-dimensional profiles. A PDB file format was provided as input to generate a profile window plot [39]. *TM-align* (https://zhanglab.ccmb.med.umich.edu/TM-align/), an online tool, was employed to predict the best alignment between two structures using both TM-score rotation matrix and dynamic programming. A TM − score < 0.2 means that there is no similarity between two protein structures [40].

### 2.5. Ligand Binding Site Prediction

COACH is a metaserver approach for prediction of protein-ligand binding sites. The server employs other comparative methods, like TM-Site and S-Site, FINDSITE, COFACTOR, and ConCavity, which are available at https://zhanglab.ccmb.med.umich.edu/COACH/ [41]. RaptorX binding site (http://raptorx.uchicago.edu/BindingSite/), a tool, was used for the prediction of ligand binding regions by submitting the FASTA format as input [42].

### 2.6. Protein Display

*Protter* (https://wlab.ethz.ch/protter/start/), a graphics open-source program, was developed to predict sequence feature annotations with experimental proteomic [43].

### 2.7. Dynamic Cross-Correlation Matrix Analysis Using Bio3d Package by RStudio Software and DynOmics Server

We determined the Dynamic Cross-Correlation Maps (DCCM) of *TMPRSS2* native and mutants using the Bio3d package by RStudio program [9]. Then, we used *DynOmics* ENM server to determinate the correlation between observed and predicted fluctuations of *TMPRSS2* native and mutants. *DynOmics* ENM, an online server, was used for computing biomolecular system dynamics of any PDB file. *DynOmics* ENM uses both elastic network models (ENMs)—the Gaussian Network Model (GNM) and the Anisotropic Network Model (ANM) [44]. Bio3d is an automated R package for the comparative analysis of biomolecular structure, sequence, analysis, and dynamic. Bio3d integrates multiple comparative methods such as principal component analysis (PCA), new ensemble difference distance matrix (eDDM) analysis, network analysis, and normal mode analysis (NMA) [45].

## 3. Results

All the reported missense variants of *the TMPRSS2* gene were retrieved from *Ensembl Genome Browser* (https://www.ensembl.org/Homo_sapiens/Gene/Variation_Gene/Table?db=core;g=ENSG00000184012;r=21:41464305-41531116). In this paper, we selected only six missense variants (rs12329760, rs781089181, rs762108701, rs1185182900, rs570454392, and rs867186402) to investigate the potential genetic susceptibility to COVID-19. For that, we used a multitier approach using different algorithms such as functional analysis of human *TMPRSS2* missense variants using SIFT, PolyPhen2.0, PROVEAN, SNAP2, PMut, and MutPred; stability analysis of mutant proteins using I-Mutant Suite, MUpro, CUPSAT, iStable, and STRUM; the implication of missense variants with conserved and exposed residues in *TMPRSS2* protein by using Clustal Omega and ConSurf tools; analysis of the effect of missense variants on protein flexibility and secondary structure using FlexPred, PredyFlexy, RaptorX property, and PredictProtein, respectively; structure analysis and comparison between tertiary structures of mutant and native proteins using Swiss Model, ModRefiner, PROCHECK, Verify3D, TM-align; and finally ligand binding site prediction using COACH and RaptorX binding site servers.

### 3.1. Functional Analysis of Human *TMPRSS2* Missense Variants

Among the six missense variants tested, five were predicted damaging (prediction score was ranged from 0 to 0.02) (Table 1). According to PolyPhen2.0, all the variants were identified as probably damaging (prediction score close to 1), while PROVEAN predicted five of the SNPs to be deleterious (G181R, R240C, P335L, G432A, and D435Y), SNAP2 predicted all of the submitted SNPs to affect protein function. When using the PMut, five of the subjected mutations were found to be disease-related (V160M, G181R, P335L, G432A, and D435Y). As presented in Table 2, MutPred analysis revealed that G181R was significantly associated with gain of a helix, loss of disulfide at C185, and the gain of ADP-ribosylation at G181 with *g* − value = 0.607 and *p* value < 0.05. It did find also that the G432A substitution induced a loss of loop, altered metal binding, the gain of disulfide linkage at C437, the gain of the catalytic site at D435, and gain of pyrrolidone carboxylic acid at Q431 with *g* − value = 0.874 and *p* value > 0.05. Finally, the D435Y substitution showed the highest *g* − value = 0.919 and a lower *p* value that was associated with a gain of disulfide linkage at C437.

### 3.2. Structural Analysis of Human *TMPRSS2* Missense Variants

#### 3.2.1. Protein Stability

I-Mutant Suite, MUpro, CUPSAT, iStable, and STRUM were used to predict the change in protein stability of *TMPRSS2*. Out of six nsSNPs submitted for stability testing, four variants (V160M, G181R, R240C, and G432A) were found as decreasing the stability of *TMPRSS2* protein according to I-Mutant Suite, MUpro, and iStable, while five out of six missense variants were predicted as destabilizing the *TMPRSS2* protein using the STRUM server. CUPSAT identified five variants (V160M, G181R, P335L, G432A, and D435Y) out of six that affect the protein stability of *TMPRSS2*. Only one variant P335L exhibited unfavorable charges in torsion angle with influence on *TMPRSS2* protein stability (Tables 3 and 4).

#### 3.2.2. Conservation Analysis of *TMPRSS2* Gene

The amino acid sequence of *TMPS2_Human transmembrane protease serine 2* protein was blasted against the UniprotKB/SwissProt in NCBI databases, and 100 sequences producing significant alignments were downloaded as Hit Table (CSV) files. Therefore, all sequences share more than 70% identity and an *E*-value equal to 0. Clustal omega was used for multiple sequence alignment (MSA). The residue identities were visualized and colored using Jalview program, according to the Clustal color scheme and the conservation score.

#### 3.2.3. Evolutionary Phylogenetic Analysis of *TMPRSS2*

**Figure 1:**
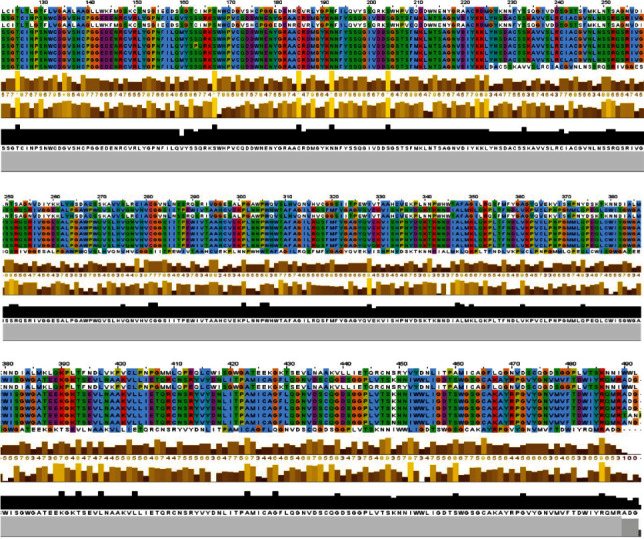
Multiple sequence alignment of the indicated *TMPS2*_*Human transmembrane protease serine 2* (Uniprot ID: O15393) sequences using Jalview.

**Figure 2:**
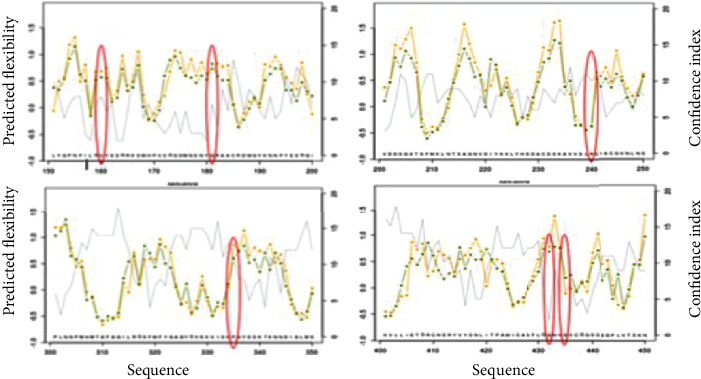
Prediction of the flexibility of the mutated and native *TMPRSS2* by PredyFlexy.

The amino acid evolutionary conservation in *TMPRSS2* protein was checked using the ConSurf server. As presented in Figures 1 and 2 and Table 2, ConSurf analysis showed that residues G181 (buried), G432, and D435 (exposed and functional) are highly conserved with an index conservation of 9 and identified less conserved amino acid residues V160 (buried) and R240 (exposed) with an index conservation of 5-6. P335 was observed to have a conservation score of 3 (variable and exposed).

#### 3.2.4. Protein Flexibility

FlexPred program was used to predict fluctuations and evaluate which amino acid residues are located in flexible or rigid regions of the *TMPRSS2* protein. It was identified that five residues valine, glycine, arginine, proline, and aspartic acid at positions 160, 181, 240, 335, and 435, respectively, were rigid, while the glycine at position 432 was predicted flexible (Table 5).

For identifying the levels of residue dynamics, we used the PredyFlexy program based on *B*-factor (relative vibrational motion) and root mean square fluctuations (RMSFs). As shown in Table 6 and Figure 2, PredyFlexy analysis showed that residues V160, G181, and P335 shared moderately and highly flexibility scores (predicted flexibility between 0 and 0.5) with a confidence index of 7-11, while the residues R240 and D435 were identified as rigid with low index scores. Then, G432 is predicted as flexible but the low confidence score (CI = 2) makes the result not reliable.

**Figure 3:**
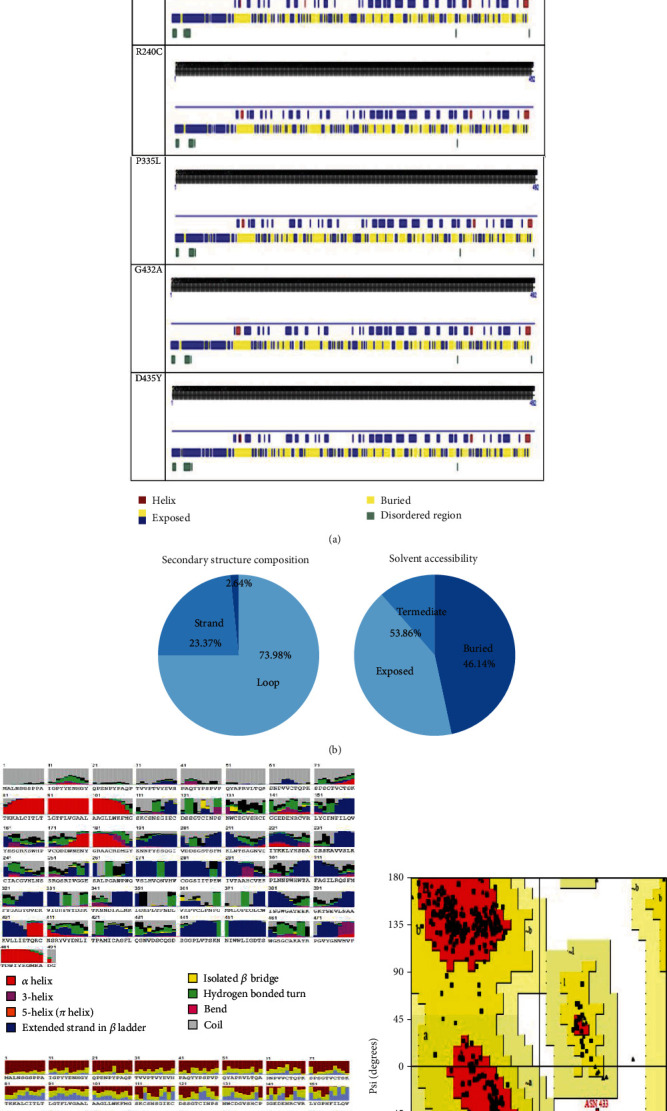
(a) Prediction of the secondary structure of the mutated and wild-type *TMPRSS2* protein. (b) Secondary structure of *TMPRSS2* protein. (c) Eight-class secondary structure of *TMPRSS2* protein by using RaptorX property server. (d) Ramachandran plot of *TMPRSS2* protein.

To determine protein secondary structure, disorder regions, and solvent accessibility of *TMPRSS2* protein, the RaptorX property was used. As exposed in Figure 3(c), 88 (17%) positions were predicted as disordered by RaptorX property; then, eight secondary structure types were identified in the *TMPRSS2* protein, such as *α* helix, 3-helix, 5-helix (ℼ helix), extended strand in *β* ladder, isolated *β* bridge, hydrogen-bonded turn, bend, and coil. Results of solvent accessibility of *TMPRSS2* protein were 27% intermedia, 46% exposed residues, and 25% buried residues (Figure 3(c)).

#### 3.2.5. Secondary Structure

To validate the solvent accessibility and protein secondary structure, we applied the PredictProtein tool. The most types of secondary structure of the *TMPRSS2* protein are the helix, buried, exposed, and disordered regions. Then, three types of protein secondary structure were identified in the *TMPRSS2* protein, which was helix 2.64% (H; includes *α*, Pi-, and 3_10-helix), *β*-strand 23.37% (E; extended strand in the *β*-sheet conformation of at least two residues length), and loop (L) 73.98%. Figures 3(a) and 3(b), display the PredictProtein analysis of the *TMPRSS2* protein (46.14% buried residues and 53.86% exposed residues).

#### 3.2.6. Modeling

The full three-dimensional structure of human *TMPRSS2* protein was not available in the Research Collaboratory for Structural Bioinformatics Protein Data Bank (RCSB-PDB, http://rcsb.org). For that, the SwissModeller group has modeled the *TMPRSS2* structure with a resolution of 1.95 Å and sequence identity equal to 98.69% was used for further analysis. The selected structures wild type and mutants were refined using ModRefiner and were validated using PROCHECK and Verify3D (Table 7). Ramachandran plot of the native protein identified 258 residues (86.9%) in favored regions, 38 residues (12.8%) in allowed regions (additional and generously allowed regions), and one residue (0.3%) in disallowed regions (Figure 3(d)). Furthermore, Verify3D analysis of the native and mutant proteins revealed that 95.38% (native) of the residues had an average 3D-1D score ≥ of 0.2, while the models (V160M, G181R, R240C, P335, G432, and D435) showed that 97.09%, 93.02%, 96.80%, 95.64%, 99.42%, and 94.19% of the residues have an average 3D-1D score ≥ 0.2.

**Figure 4:**
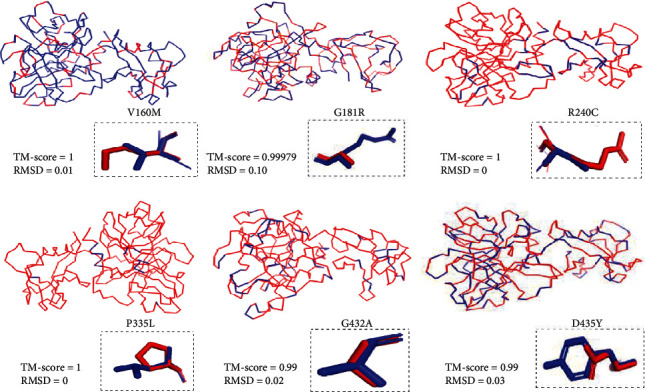
Superimposition of mutant protein structures (in blue) on the wild-type *TMPRSS2* (in red) using PyMOL program.

Besides, structural similarities between the wild-type and mutant structures were performed using TM-align tool based on TM-score to assess the topological similarity of two proteins and the RMSD (Root Mean Square Deviation) to measure the distance between the backbones of the superimposed protein structures. The RMSD values for all missense variants were significant (RMSD > 0.15), suggesting dissimilarity between wild-type and mutant models (Figure 4, Table 8).

#### 3.2.7. Ligand Binding Site Prediction

To identify ligand binding sites in the *TMPRSS2* protein, we used RaptorX binding and COACH servers. According to the RaptorX binding tool, the largest pocket multiplicity was 55 (pocket multiplicity > 40 indicates a true prediction of the pocket), which binds to the residues H256, **D435**, S436, C437, Q438, G439, S441, T459, S460, W461, G462, S463, and G464 (Table 9).

According to the COACH server, **D435** was predicted as a binding residue. The detailed results of COACH are shown in Table 10.

#### 3.2.8. Protein Display

**Figure 5:**
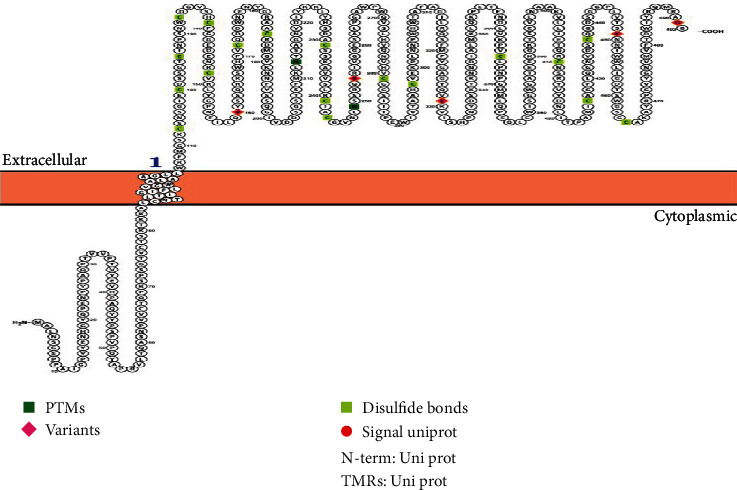
*TMPRSS2* protein localization visualized using the Protter sever.

The topology prediction was shown by the Protter server; the figure illustrates a long cytoplasmic N-terminus and suggests that the *TMPRSS2* protein was located mostly at the extracellular part of the cell membrane. Then, the five amino acids (orange color) represent the predicted variants such as V160, S254, E331, K451, and D491 (Figure 5).

#### 3.2.9. Dynamic Cross-Correlation Matrix Analysis Using Bio3d Package by RStudio Software and DynOmics Server

**Figure 6:**
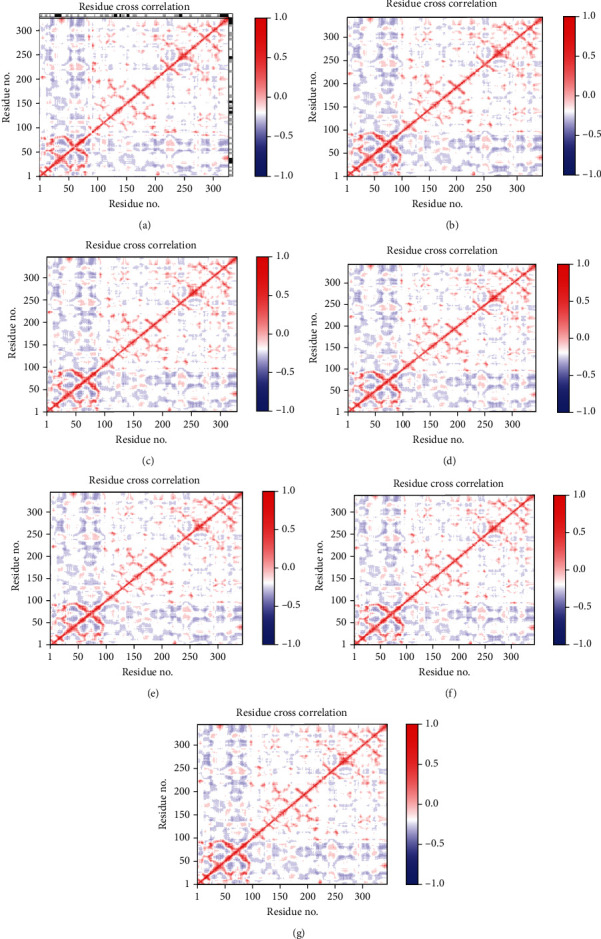
Dynamic Cross-Correlation Map (DCCM) analysis of *TMPRSS2* native and mutants: (a) wild type, (b) V160M, (c) G181R, (d) R240C, (e) P335L, (f) G432A, and (g) D432Y.

DCCM was done to comprehend the correlated communications between residues. The result showed that as compared with the wild type, the V160M, G181R, R240C, P335L, G432A, and D435Y variants decreased the degree of positive (red color) and negative (blue color) correlations observed in the *TMPRSS2* native, despite the fact that no significant correlation in the movement of residues has been remarked in the Dynamic Cross-Correlation Matrix analysis (Figure 6, Table 11).

## 4. Discussion

The transmembrane serine protease 2 (*TMPRSS2*) plays a crucial role in human cell entry of a diverse range of viruses including SARS-CoV-2 [2]. Strikingly, a recent investigation by Hou et al. found six deleterious variants such as p. Val 160Met, p. Gly181Arg, p. Arg240Cys, p. Pro335Leu, p. Gly432Ala, and p. Arg435Tyr in the *TMPRSS2* gene, which are demonstrated as somatic mutations in different cancer databases and also suggest explanations for genetic susceptibility to COVID-19 [11]. This analysis reported that *TMPRSS2* variants were probably associated with susceptibility to SARS-CoV-2 [11]. So, in this report, we look for these six missense variants (V160M, G181R, R240C, P335L, G432A, and D435Y) which previously might be important risk factors associated with COVID-19 susceptibility. The current study might also be helpful to understand the effect of those variants on *TMPRSS2* structure, function, and stability. A series of in silico prediction analyses were used for the functional and structural annotations of human *TMPRSS2* missense variants like SIFT, PolyPhen2.0, PROVEAN, SNAP2, PMut, MutPred2, I-Mutant Suite, MUpro, iStable, CUPSAT, and STRUM, respectively, which were utilized to find out the most deleterious variants of *TMPRSS2* and to evaluate their effects on *TMPRSS2* function, structure, and stability.

From our functional analysis of human *TMPRSS2* missense variants, SIFT predicted five of total variants are deleterious; these five variations were predicted deleterious by PROVEAN (except for V160M), SNAP2, and PMut (except for R240C). Protein stability is essential for understanding the relationship between protein structure and function [46]. A total of six variants tested were identified decreasing the stability of *TMPRSS2* by all algorithms for V160M and G181R and by at least three tools for the rest (R240C, P335L, G432A, and D435Y), by analyzing all missense variants through different servers. The six nsSNPs are potentially damaging. ConSurf analysis results showed that variants at positions G181R, G432A, and D435Y were in the highly conserved region and confirmed by MutPred2 to have crucial alterations on the *TMPRSS2* protein. The prediction of posttranslational modification sites (PTMs) is one of the important characteristics for understanding different biological processes such as the cell signalling state, localization, and interactions. It can also be essential for the study of diseases or for development of drugs [47]. Therefore, the R240, P335, G432, and D435 residues identified PTMs for proteolytic cleavage and ADP-ribosylation. Flexibility is one of the most essential criteria related to protein functions. Herein, we used FlexPred and PredyFlexy to determine conformational changes and to comprehend dynamic system of *TMPRSS2*. Variants R240C and D435Y were predicted to be in a relatively rigid region, while G432A was defined as a flexible area. We have also investigated the secondary structure of native and mutants by identifying disordered regions in *TMPRSS2* using PredictProtein and RaptorX property. Compared to the native structure of *TMPRSS2* protein, 5 disordered regions were formed due to V160M and P335L variants, since this can change the function of *TMPRSS2* because disordered regions are dynamically flexible. Prediction of three-dimensional structures of *TMPRSS2* models is necessary for the validation of structural changes. Therefore, the three-dimensional structure of the *TMPRSS2* native and mutants was generated using 7 meq as a template from the SwissModeller group and refined by using ModRefiner. Quality checking of SwissModel constructed models was done by using PROCHECK and Verify3D. Ramachandran plot analysis showed that all models of *TMPRSS2* (wild-type and mutants) were of good quality and can be used for further study; then, quantitative assessment was done by using the TM-align tool for comparing native and mutant proteins by calculating RMSD values and TM-score. All RMSD values were significant (RMSD > 0.15). The highest RMSD value 0.58 was scored by the variant R240C, while the lowest 0.44 was scored by the variant G432A. Besides, we used the RaptorX binding site and COACH servers for finding sites of further variants. However, two residues D435 and P335 were identified to be implicated in ligand binding site interactions of ligands with the *TMPRSS2* protein. Consequently, our results give the clue that V160M, G181R, R240C, P335L, G432A, and D435Y can be the most significant variants in the human *TMPRSS2* gene and may influence stability, structure, function, and interaction of ligands with the *TMPRSS2* protein.

To date, various in silico analyses have been made using different bioinformatics tools to identify and predict *TMPRSS2* gene host polymorphism against SARS-CoV-2. As our results show, a study by [48] has shown that the *TMPRSS2* p. Val160Met polymorphism was associated with SARS-CoV-2 infectivity. A recent investigation by Asselta et al. reported the existence of some *TMPRSS2* polymorphisms, namely, rs2070788, rs9974589, and rs7364083. These variants showed a significant association between these SNPs and the SARS-CoV-2 infectivity [40]. Another study by Irham et al. (2020) demonstrated that some variants of *TMPRSS2*, namely, rs2070788, rs383510, rs464397, and rs469390, might affect the expression of *TMPRSS2* in some many tissues and consequently were probably associated with SARS-CoV-2 infectivity [51].

Overall, this in silico analysis gives an interesting insight into the role of the *TMPRSS2* variants in susceptibility to SARS-CoV-2 infection. The analysis consortium would also involve researchers and scientists in the future to confirm the selected mutations (V160M, G181R, R240C, P335L, G432A, and D435Y) as candidate variants. In the future, it should be noted that further in silico analysis and laboratory experiments must be combined for more justifying such important results.

## 5. Conclusion

Overall, we conclude that rs12329760 (V160M), rs781089181 (G181R), rs762108701 (R240C), rs1185182900 (P335L), rs570454392 (G432A), and rs867186402 (D435Y) are the most significant variants. All six nsSNPs were predicted to alter protein function and stability. Most of them are highly conserved (V160M, G181R, G432A, and D435Y) and comprise posttranslational modification sites (PTMs) (R240C, P335L, G432A, and D435Y). D435 was identified as a ligand-binding site that may interfere in the binding interactions of the *TMPRSS2* protein. In this *in silico* analysis, for the first time, we tested the effect of those missense variants on *TMPRSS2* structure, stability, and function by using various bioinformatics algorithms that may serve an important role in SARS-CoV-2 infection.

## Figures and Tables

**Table 1:** Missense variants identified to be deleterious or damaging using different algorithms.

SNP ID	Amino acid change	SIFT		PolyPhen2.0		PROVEAN		SNAP2		PMut
		Score	Prediction	Score	Prediction	Score	Prediction	Score	Prediction	
rs12329760	V160M	0.01	D	0.997	P. D	-1.891	N	95	E	Dis
rs781089181	G181R	0.06	T	1.000	P. D	-6.057	Del	45	E	Dis
rs762108701	R240C	0.01	D	1.000	P. D	-5.224	Del	63	E	N
rs1185182900	P335L	0.02	D	0.985	P. D	-7.515	Del	39	E	Dis
rs570454392	G432A	0.00	D	1.000	P. D	-5.631	Del	63	E	Dis
rs867186402	D435Y	0.00	D	1.000	P. D	-7.975	Del	74	E	Dis

Legend: D: damaging; T: tolerated, P. D: probably damaging; Del: deleterious; N: neutral; E: effect; Dis: disease.

**Table 2:** Prediction of effect of missense variants on phylogenetic conservation, phenotypic analysis, and posttranslational modification sites in human *TMPRSS2* protein.

SNP ID	Variant	Posttranslational modifications (PTMs) by ModPred	Phylogenetic conservation	Predicted effect by MutPred
rs12329760	V160M	—	6, B	—
rs781089181	G181R	—	9, B	Loss of loop
				Altered transmembrane protein
				Gain of helix
				Loss of disulfide linkage at C185
				Gain of ADP-ribosylation at G181

rs762108701	R240C	Proteolytic cleavage	5, E	—
		ADP-ribosylation		

rs1185182900	P335L	Proteolytic cleavage	3, E	—

rs570454392	G432A	Proteolytic cleavage	9, E, F	Loss of relative solvent accessibility
				Loss of loop
				Altered transmembrane protein
				Altered metal binding
				Gain of disulfide linkage at C437
				Gain of catalytic site at D435
				Gain of pyrrolidone carboxylic acid at Q431

rs867186402	D435Y	Proteolytic cleavage	9, E, F	Altered transmembrane protein
				Altered ordered interface
				Altered metal binding
				Loss of relative solvent accessibility
				Loss of catalytic site at G439
				Gain of disulfide linkage at C437
				Gain of pyrrolidone carboxylic acid at Q43
				Gain of sulfation at D435

**Table 3:** Effects of mutation on protein stability by I-Mutant, MUpro, iStable, and STRUM.

SNP ID	Amino acid variant	I-Mutant	MUpro	iStable	STRUM
rs12329760	V160M	Decrease	Decrease	Decrease	Destabilizing
rs781089181	G181R	Decrease	Decrease	Decrease	Destabilizing
rs762108701	R240C	Decrease	Decrease	Decrease	Destabilizing
rs1185182900	P335L	Decrease	Increase	Increase	Destabilizing
rs570454392	G432A	Decrease	Decrease	Decrease	Stabilizing
rs867186402	D435Y	Increase	Decrease	Increase	Destabilizing

**Table 4:** Missense variant analysis by CUPSAT tool.

SNP ID	Amino acid variant	Stability	Torsion	Predicted ∆∆*G* (kcal/mol)
rs12329760	V160M	Destabilizing	Favorable	-3.39
rs781089181	G181R	Destabilizing	Favorable	-0.57
rs762108701	R240C	Stabilizing	Favorable	0.75
rs1185182900	P335L	Destabilizing	Unfavorable	-2.39
rs570454392	G432A	Destabilizing	Favorable	-6.86
rs867186402	D435Y	Destabilizing	Favorable	-1.68

**Table 5:** Prediction of *TMPRSS2* flexibility using FlexPred server.

Position	Residues	S_LBL ((R) rigid or flexible (F) label)	S_PRB (probability of flexible (F) label)
160	VAL	R	0.4874
181	GLY	R	0.6059
240	ARG	R	0.5174
335	PRO	R	0.5862
432	GLY	F	0.7747
435	ASP	R	0.6696

**Table 6:** Flexibility analysis by PredyFlexy.

	RMSF	*B*-factor	Confidence index (CI)
V160M	0.687	0.574	8
G181R	0.725	0.824	7
R240C	-0.359	-0.375	10
P335L	0.854	0.601	11
G432A	0.788	0.690	2
D435Y	-0.105	0.195	6

**Table 7:** *TMPRSS2* structure validation using Verify3D and PROCHECK servers.

	Verify3D	PROCHECK		
	% of amino acid scored > 0.2 in the 3D/1D profile	Favored region	Allowed region	Disallowed region
Native	93.02	86.9% (258)	12.8% (38)	0.3% (1)
V160M	97.09	88.9% (264)	10.1% (30)	1.0% (3)
G181R	93.02	89.3% (266)	10% (30)	0.7% (2)
R240C	96.80	89.9% (267)	9.4% (28)	0.7% (2)
P335L	95.64	90.6% (270)	8.7% (26)	0.7% (2)
G432A	99.42	88.6% (264)	10.7% (31)	0.7% (2)
D435Y	94.19	87.2% (259)	11.8% (35)	1.0% (3)

**Table 8:** Structure alignment comparing mutant models and native *TMPRSS2* proteins.

Position	Variant	TM-align score server		
		Align	RMSD	TM-score
160	V160M	7 meq1.A	0.51	0.99435
181	G181R	7 meq1.A	0.51	0.99431
240	R240C	7 meq1.A	0.58	0.99304
335	P335L	7 meq1.A	0.53	0.99403
432	G432A	7 meq1.A	0.44	0.99590
435	D435Y	7 meq1.A	0.48	0.99510

**Table 9:** Ligand binding site prediction of the *TMPRSS2* protein by RaptorX binding.

Pocket	Multiplicity	Ligand	Binding residues
**1**	55	QGG, SO4, BEN, TFA, CH2	H296, **D435**, S436, C437, Q438, G439, S441, T459, S460, W461, G462, S463, G464
**2**	19	SO4	Y416, S463, G464
**3**	19	SO4	D338, N343, N344
**4**	14	SO4	**P335**, W483, Q487
**5**	11	SO4, PG4	Q276, N277, L302

The bold values show the residues included in the current study.

**Table:** (a) COACH

*C*-score	Cluster size	Name of ligand	Residue number
**0.82**	1440	T87	296, 340, 341, 342, **435**, 436, 437, 438, 441, 459, 460, 462? 463, 464, 465, 472.
**0.05**	123	PEPTIDE	275, 280, 281, 296, 297, 300, 301, 308, **435**, 436, 437, 438, 439, 441, 459, 460, 461, 462, 463, 464, 472.
**0.03**	93	PEPTIDE	260, 261, 263, 264, 265, 266, 268, 269, 358, 359, 362, 363, 364, 365, 377, 378, 380, 399, 401, 429, 447, 448, 451, 452, 453.
**0.02**	77	PEPTIDE	274, 278, 311, 317, 318, 319, 320, 322, 325.
**0.02**	55	PEPTIDE	265, 266, 267, 268, 269, 357, 359, 362, 363, 364, 365, 380, 399, 452, 453.
**0.02**	52	PEPTIDE	274, 277, 279, 280, 296, 309, 317, 318, 319, 320, 325, 327, 340, 393, **435**, 436, 438, 439, 440, 441, 460, 461, 462, 464, 472.
**0.01**	27	PEPTIDE	274, 278, 279, 317, 318, 319.
**0.01**	35	CA	314, 316, 317, 318, 319, 320, 323.
**0.01**	21	PEPTIDE	265, 266, 267, 268, 269, 288, 355, 356, 357, 359, 361, 362, 363, 364, 365, 380, 453.
**0.00**	4	SO4	367, 368, 369, 454.

**Table:** (b) TM-Site

*C*-score	Cluster size	Name of ligand	Residue number
**0.50**	113	III, 0G6, 0GJ	296, 342, **435**, 436, 437, 438, 439, 441, 459, 460, 461, 462, 463, 464, 465, 472.
**0.24**	23	III, C3A, SO4	275, 280, 281, 296, 297, 300, 301, 308, **435**, 436, 437, 438, 439, 441, 459, 460, 461, 462, 463, 464, 472.
**0.19**	29	III	263, 264, 265, 266, 268, 269, 358, 359, 360, 362, 363, 364, 365, 376, 377, 378, 380, 401, 429, 447, 448, 450, 451, 452, 453.
**0.19**	22	III, GSH, BR	265, 266, 268, 269, 357, 359, 362, 363, 364, 365, 380, 399, 452, 453.
**0.16**	7	III, ZN, IOD	274, 277, 278, 279, 280, 296, 301, 309, 311, 317, 318, 319, 320, 325, 340, 341, **435**, 436, 438, 439, 441, 460, 461, 462, 464, 472.

**Table:** (c) S-Site

*C*-score	Cluster size	Name of ligand	Residue number
0.38	752	III, BEN, UUU	280, 281, 296, 297, 300, 341, 342, 418, 435, 436, 437, 438, 439, 440, 441, 459, 460, 461, 462, 463, 464, 472, 473, 474.
0.14	80	III, UUU, GSH	260, 263, 264, 265, 266, 267, 268, 269, 288, 355, 356, 357, 358, 359, 360, 361, 362, 363, 364, 365, 372, 376, 377, 378, 379, 380, 401, 429, 447, 448, 451, 452, 453.
0.13	98	III, CA, EDO	274, 276, 277, 278, 279, 309, 311, 314, 316, 317, 318, 319, 320, 323, 324, 325, 327.
0.11	27	NA, CA, ZN	413, 416, 429, 430, 431, 433, 463, 466, 467, 468, 469, 470, 471, 473.
0.10	13	BGC, SO4, CA	372, 373, 375, 404, 405, 406, 407, 408, 409, 410, 421, 422, 423, 424, 425, 426, 456, 476.

**Table:** (d) COFACTOR

*C*-score	Name of ligand	Residue number
0.51	PEPTIDE	296, 337, 340, 342, 389, 419, **435**, 436, 437, 438, 439, 441, 460, 461, 462, 463, 464, 465, 472.
0.45	T76	296, 436, 441, 459, 460, 461, 462, 464, 465, 472, 473.
0.42	BM2	296, 341, 342, **435**, 438, 441, 460, 461, 462, 463, 464.
0.26	PEPTIDE	296, **435**, 436, 437, 461, 462, 464, 472.
0.25	PEPTIDE	296, 389, 390, 441, 460, 462, 464.

**Table:** (e) FINDSITE

*C*-score	Cluster size	Name of ligand	Residue number
0.70	320	Site 1	296, 342, 418 **435**, 436, 437, 438, 441, 459, 461, 462, 463, 464, 465, 472, 474.
0.10	46	Site 2	272, 274, 276, 277, 279, 309, 311, 317, 318, 319, 320, 324, 325, 327, 393.
0.04	16	Site 3	265, 266, 267, 268, 269, 285, 288, 355, 375, 359, 362, 363, 365, 380, 452, 453.
0.03	14	Site 4	299, 302
0.01	4	Site 5	338, 339

**Table:** (f) ConCavity

*C*-score	Name of ligand	Residue number
**0.45**	Cavity 1	280, 296, 297, 341, 342, 345, 381, 402, 416, 419, 420, 427, 428, 429, 434, **435**, 436, 437, 438, 439, 440, 441, 445, 458, 459, 460, 461, 462, 464, 465, 467, 470, 471, 472, 473, 474.
**0.30**	Cavity 2	267, 270, 271, 272, 279, 282, 317, 383, 384, 397, 439, 440.
**0.21**	Cavity 3	268, 269, 270, 271, 285, 288, 289, 291, 310, 312, 313, 327, 328, 349, 351, 355, 360, 361, 362.

The bold values show the residues included in the current study.

**Table 11:** Correlation between observed and predicted fluctuations of *TMPRSS2* native and mutants.

*TMPRSS2* structures	Native	V160M	G181R	R240C	P335L	G432A	D435Y
Correlation between observed and predicted fluctuations	0.8	0.69	0.70	0.69	0.72	0.69	0.69

## Data Availability

All data used in this paper are included within the article.
